# Research progress on digital health literacy of older adults: A scoping review

**DOI:** 10.3389/fpubh.2022.906089

**Published:** 2022-08-05

**Authors:** Xinxin Wang, Wei Luan

**Affiliations:** ^1^Shanghai Jiao Tong University School of Nursing, Shanghai, China; ^2^Department of VIP Service, Renji Hospital, School of Medicine, Shanghai Jiao Tong University, Shanghai, China

**Keywords:** digital health literacy, e-health literacy, older adults, older people, scoping review

## Abstract

With the rapid development of digital health today, the lack of digital health literacy in older adults is an urgent problem. It is crucial that older adults adapt to the digital reform in medical treatment, pension, health management, and other fields. Therefore, we reviewed the current development status of digital health literacy among older adults. A total of 47 articles were included in this scoping review. Our findings revealed that research on digital health literacy in older adults is still in its infancy. Further development is warranted especially in terms of assessment tools and intervention methods.

## Introduction

With the rapid development of modern science and technology, the achievements of digital technologies such as artificial intelligence, virtual and augmented reality, and machine learning have been continuously applied in healthcare services (1). While the quality of healthcare products and services has improved, the ways in which older adults acquire and share health knowledge is also changing (2). In China, 95.09% of older adults believe that it is necessary to learn to browse the Internet after the initial stages of the COVID-19 pandemic, and 93.36% suppose that they can learn to use a smartphone (3), which reflects their strong desire to use digital technology. Therefore, to arouse enthusiasm of older adults in health management and make digital devices and software more accessible for use, it is also important to improve the digital health literacy (DHL) of older adults. DHL is an extended concept of health literacy, which refers to the ability of individuals to acquire, process, communicate, and understand health information and services, make effective health decisions, and promote and improve individual and collective health in the context of the use of digital information and technologies (4).

According to the definition provided by Norman and Skinner (5), eHealth Literacy (eHL) is “the ability to seek, discover, evaluate, and appraise eHealth information, and apply the acquired knowledge to solve health problems” (5). Moreover, Norman pointed out that with the continuous development of new technologies and environmental changes, the way in which health knowledge spreads will evolve with the corresponding environmental changes (6). The development of science and technology in recent years has a significant impact on individual's medical procedures, health management methods, and community health services. Although many scholars have considered the impact of technological development on the concept of eHL. However, while they have tried to update the concept of eHL, they still use the term eHL (7–9). In 2012, the concept of DHL was first mentioned (10). Compared with eHL, DHL measures focus on interactivity on the Web, including adding self-generated content and protecting privacy (11). Under the framework of DHL, health knowledge is presented to individuals more attractively, by providing continuous, dynamic, and highly personalized health management solutions, improving personal health management capabilities while focusing on individual health and living a healthier life (1).

Prior literature shows that age, gender, educational attainment, marital status, credibility of Internet health information, experience and more were identified as modifiable factors related to eHL in older adults (12–14). Kim et al. (15) and Karnoe et al. (16) summarized the assessment tools for eHL. The systematic review of Oh SS et al. focused on the evaluation tools of eHL in older adults (17). Jacobs RJ et al. outlined eHealth interventions to improve health literacy, and the review results show that computer-based applications were the most common intervention methods (18). Choukou MA et al. summarized how e-services implemented in vulnerable populations improved DHL during the COVID-19 pandemic and identified the barriers and facilitators for their implementation (19). However, most reviews still use the concept of eHL and they do not distinguish between eHL and DHL. While eHL and DHL are often used interchangeably, eHL measurement is distinguished by focusing on online health information gathering (11). As the concept of digital literacy has evolved in recent years, so has that of DHL. Therefore, this scoping review includes studies that apply both eHL and DHL concepts, considering eHL as the predecessor of DHL.

### Purpose

This review aims to describe and synthesize published research related to DHL among older adults. The focus is on research findings related to the influencing factors, impacts, assessment tools, and intervention methods. The questions that guided the review were: (1) What factors affect DHL in older adults? (2) How does DHL affect older adults? (3) Which tools have been used to assess DHL among older adults? (4) What are the existing methods to improve the DHL of older adults?

## Methods

A scoping review intends to examine the scope and nature of existing research on a topic or issue, determine the value of conducting a comprehensive systematic review, and identify gaps in the existing research (20). A scoping review methodology is based on the framework outlined by Arksey and O'Malley (21). Scoping reviews use descriptive summaries and inductive analysis to summarize research findings. This review aims to provide a comprehensive summary of the existing relevant literature, and therefore does not perform a critical appraisal of included studies.

### Search

Articles were identified by searching four databases: Web of Science (All Databases), PubMed, Embase.com, and Chinese database CNKI. The databases were searched in January 2022 and June 2022. A librarian at the University was consulted for assistance with the search. The following search terms were used: digital health literacy; eHealth literacy; e-health literacy; old^*^; old people; older; older people; older adult; elder; elder people; elders; elderly people; elder adults; aged; aged people; aged person; aging. Secondary searches included the following terms: computer literacy; online health literacy; electronic health literacy; health information literacy; health information seeking; health information searching; senior; baby boomer; retiree^*^. Inclusion criteria were: (1) Chinese and English literature published between 2011 and 2021. (2) Literature related to DHL or eHL. (3) The participants were older adults (age ≥ 65) or belonged to a subgroup of older adults. Exclusion criteria were: (1) Reviews, books, letters to the editor, and abstracts of speeches. (2) The participants were adults, and there was no subgroup of older adults.

### Search outcome

**Figure 1:**
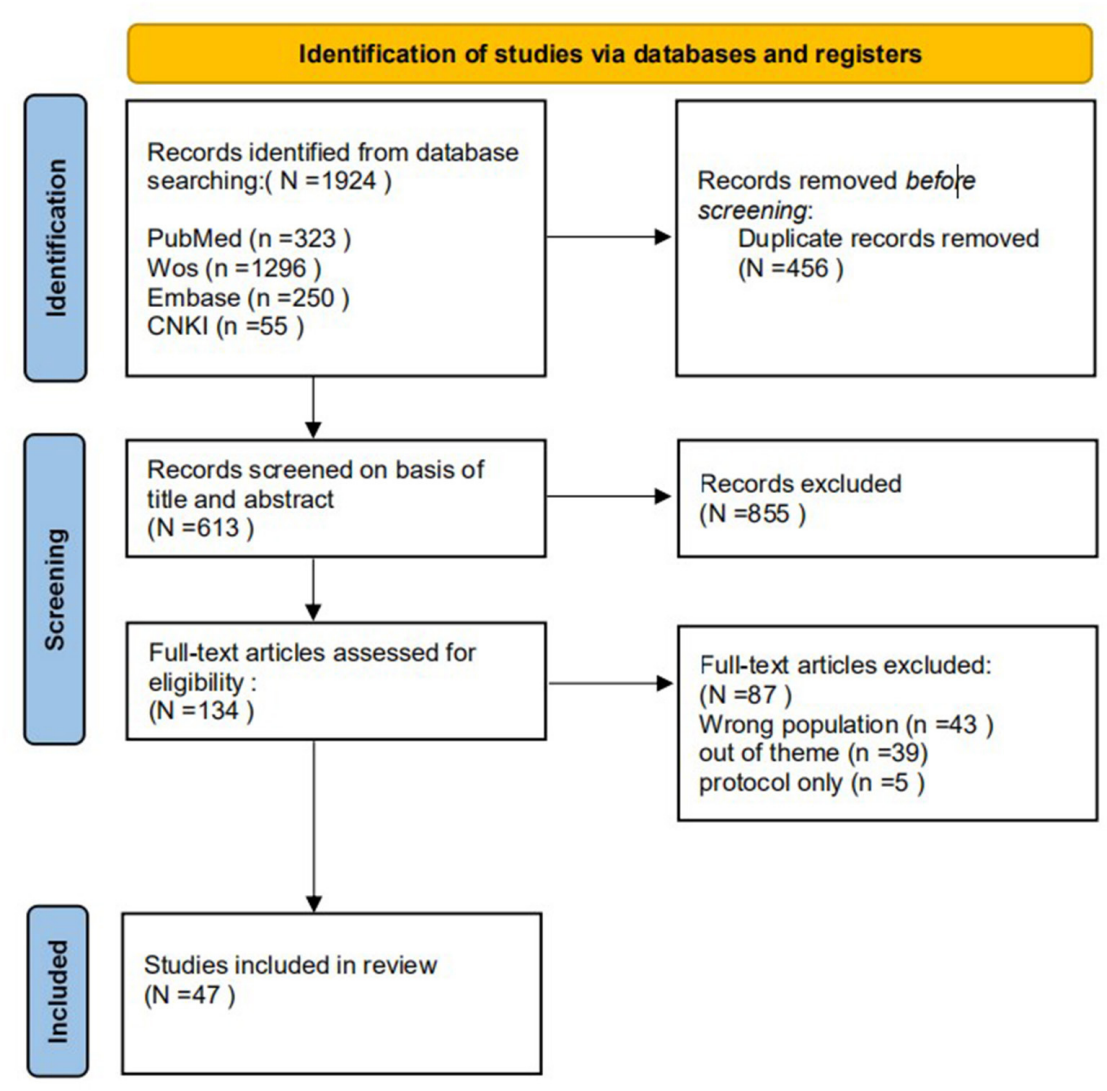
Flowchart of the literature screening process.

The initial and secondary search yielded 1,924 articles. The literature search results were reviewed, and duplicate results were excluded using Endnote X9, leaving 1,468 articles. According to the inclusion and exclusion criteria of the study, two authors independently scrutinized the titles and abstracts of the articles, leaving 613 articles. If two reviewers had doubts, the full version was analyzed independently. Disagreements between the reviewers were solved by a third reviewer. A total of 134 full-text articles were assessed for eligibility. After screening the full-text articles, 47 articles were finally included [Figure 1 (22)].

## Results

### Influencing factors

#### Socio-demographic factors

Several studies showed that gender, age, place of residence, education level, marital status, socioeconomic status, pension methods, and type of medical insurance are the main factors affecting the DHL of older adults (23–32). Those who were younger, had a higher level of education, and a higher socioeconomic status tended to have higher DHL.

#### Factors related to digital equipment

Factors such as whether older adults own digital devices (23, 28), the frequency of using digital devices (31, 33), and the range of Internet activities (31) will also affect their DHL. Older adults who own digital devices and have a high usage rate are more likely to have high DHL.

#### Social support factors

Older people's confidence in their DHL often depends on others. If someone in the family is proficient in digital technology and can effectively share health information, they are able to manage their health together (34). Meanwhile, a library or community support also plays a positive role in improving health behaviors and outcomes in older adults (35).

#### Psychological factors

Older people with more positive attitudes toward health knowledge (23, 35), higher interest in digital technology, and confidence in managing their health through digital devices have higher self-rated DHL scores (24, 36, 37) (Table 1).

**Table 1:** Influencing factors of DHL among older adults.

**Authors, publication year, country**	**Objective**	**Method**	**Theory**	**Assessment tools**	**Results**	**Limitations**
De Santis et al. (24) Germany	To investigate the attitudes toward and the use of digital technologies for health-related purposes using a nationwide survey	Cross-sectional study	eHL	eHEALS, Self-designed questionnaire	A higher perceived eHL score was associated with younger age, higher household income, and more education.	The data were collected using a single source (quantitative survey) and relied on self-reports.
Cui et al. (26) China	To explore the relationships among social capital (structural and cognitive social capital), eHL, and the health behaviors of elderly people.	Cross-sectional study	eHL	Shortened eHEALS, Social Capital Scale (CSSCS), Health-Promoting Lifestyle Profile (HPLP)	Social capital and eHL were significantly correlated with health behaviors, and social capital and structural social capital were significantly correlated with eHL.	1. The study did not analyse its internal structure, the uncertainties about the dimensions of the eHEALS can be a limitation 2. Only some elderly people from Jinan City were investigated.
Li et al. (32) China	To examine the association between eHL and health-related quality of life (HRQoL).	Cross-sectional study	eHL	eHEALS, Short-Form Health Survey (SF-12)	eHL was significantly positively associated with health-promoting behaviors, and health-promoting behaviors were associated with HRQoL.	1. Selection bias. 2. Self-report measures, which may have led to some information bias 3. The analytic sample is not representative of all older adults.
Berkowsky et al. (31) United States	To identify disparities in eHL among older adults aged 65 + residing in California, USA	Cross-sectional study	eHL	eHEALS	The strongest and most consistent predictors of eHL include education, frequency of Internet use, and breadth of Internet activities regularly performed.	1. The analytic sample is not representative of all older adults. 2. The participants filled in the questionnaire online, the sample is likely more technologically proficient compared to the general population.
Papp-Zipernovszky et al. (33) Hungary	To explore these generational differences as related to self-perceived eHL and health care system utilization.	Cross-sectional study	eHL	eHEALS, Self-perceived gain in empowerment scale	The study found significant differences among the generations in eHL as well as in the self-perceived gain in empowerment. The ones with higher eHl scores report better subjective health status.	The analytic sample is not representative.
Lin et al. (35) China	To examine the eHL, health knowledge, health behavior of a population of older Chinese adults, and the impact of using library or community activities for health information seeking.	Cross-sectional study	eHL	eHEALS, Self-designed questionnaire	Health behavior had a significant relationship with eHL and health knowledge. Both eHL and health knowledge showed a significant positive relationship with using the library or community activities for health information.	The analytic sample is not representative.
Liu et al. (38) China	To investigate the status of eHL of rural elderly and analyze its influencing factors.	Cross-sectional study	eHL	eHEALS, Self-designed questionnaire	One-way ANOVA showed that: male, 60–69 years old, high school education, secondary school, monthly income of >2000 yuan, married, other living conditions, chronic diseases, internet access, and Internet use frequency all had effects on the score of electronic health literacy of rural elderly.	The analytic sample is not representative.
Lee et al. (27) South Korea & United States	To compare individual situations and structural factors that support the use of ICT among older adults in the US and South Korea.	Cross-sectional study, semi-structured, face-to-face interviews	eHL	eHEALS, Self-designed questionnaire, Attitudes Toward Computers/Internet Questionnaire (ATCIQ, Computer Anxiety Scale (CAS), Internet Social Capital Scales (ISCS)	In both groups, factors affecting eHL included educational levels and confidence in using ICT.	The analytic sample is not representative.
Cherid et al. (28) Canada	To identify the current level of technology adoption, health, and eHL among older adults with a recent fracture.	Cross-sectional study	eHL	eHEALS, Self-designed questionnaire, Single Item Literacy Screener (SILS)	eHEALS scores were similar among men and women, and between younger age group categories, but lower in the oldest age group (*p* <0.05).	1. The analytic sample is not representative. 2. Selection bias.
Yang et al. (36) South Korea	To compare the factors associated with adults' eHL.	Cross-sectional study (Online survey for young adults and a face-to-face survey for older adults)	eHL	eHEALS, Self-designed questionnaire	Older adults held more positive attitudes toward internet health information than young adults, eHL levels were comparable. Attitude toward internet health information was a significant predictor of eHL in both groups, and age was only a predictor among young adults.	The study focuses on comparison of eHL among young and elder adults, without delving into the influencing factors.
Magsamen-Conrad et al. (34) United States	To investigate how do middle-aged and older adults use technology to seek health information and communicate with others about health and technology? What role does literacy play in the process of using technology to seek health information?	Semi-structured interview	eHL	/	Findings suggest that health can be co-managed if at least one person in a family unit is technologically “savvy” and able to effectively share health information.	The study only interviewed one member of a family unit and did not collect a dyadic perspective on health-information co-management.
Arcury et al. (23) United States	To examine Internet use and eHL among older adults (aged 55 + years) who were patients at clinics serving low-income populations.	Cross-sectional study	eHL	eHEALS, Self-designed questionnaire	eHL was associated with computer characteristics (number of e-devices, computer stress), and health knowledge and attitudes (medical decision making, health information sources). In multivariate analysis, computer stress maintained a significant inverse association with eHL.	1. The participation rate is limited. These factors limit the generalizability of the results. 2. This survey did recruit a large, multiethnic, low-income sample that included both rural and urban patients.
Zhou et al. (30) China	To investigate the current situation and influencing factors of e-health literacy among community older adults.	Cross-sectional study	eHL	eHEALS, Self-designed questionnaire	Socioeconomic status, family members and professors using the Internet to find health resources are important factors affecting the eHL of the elderly in the community	The analytic sample is not representative.
Cajita et al. (37) United States	To examine factors that influence intention to use mHealth among older adults with heart failure.	Cross-sectional study	eHL	eHEALS, Self-designed questionnaire, Adapted Technology Acceptance Model (TAM) scale	Perceived financial cost and eHL were not significantly associated with intention to use mHealth.	1. Most of the study's participants (94%) were “younger” older adults (65–79 years); hence, our findings may not be generalizable to the oldest members of the population with HF. 2. The study sample tended to include those with higher education and income than the average American older adult
Tennant et al. (29) United States	To explore the extent to which sociodemographic, social determinants, and electronic device use influences eHL and use of Web 2.0 for health information among baby boomers and older adults.	Cross-sectional study	eHL	eHEALS, Self-designed questionnaire, Adapted Technology Acceptance Model (TAM) scale	Respondents reporting use of Web 2.0 reported greater eHL than those who did not use Web 2.0. Younger age, more education, and use of more electronic devices were significantly associated with greater eHL.	The landline sampling method that was employed, which excluded over one-third of the state population that owns only a mobile phone

### The impacts of DHL

When exploring the relationship between an eHL health-promoting lifestyle, and health cognition in the Chinese older adults, Li et al. proposed that a health-promoting lifestyle is related to eHL and cognitive health (32), and Cui et al. found that eHL was an important mediating factor for older adults' structural social capital and health behaviors (26). Liu et al. stated that the eHL of older adults can directly affect their quality of life and indirectly affect it by influencing life satisfaction (39). Seçkin et al. discovered that in their sample of older adults the eHL and electronic confidence measures were significant predictors of a positive health perception index (40). Ernsting C proposed that eHL is necessary for the successful use of health apps and should be fully considered in designing health education strategies (41). Lin et al. pointed out that eHL has direct and indirect effects on medication adherence and quality of life (38). In addition, in the context of the COVID-19 pandemic, some scholars have emphasized that older adults' sense of coherence has a direct negative impact on anxiety and plays a mediating role in the relationship between anxiety and DHL or financial satisfaction (42, 43). According to the health empowerment theory, enhancing health empowerment requires identifying and recognizing personal and social background resources. For older adults isolated at home alone, eHL and social support can predict their self-care behaviors, which can be used to promote and sustain self-care practices (44).

### Assessment tools

Since Norman and Skinner developed the first eHL assessment scale in 2006, an increasing number of assessment scales have been created. With the continuous development of science and technology, the core of assessment gradually shifted from eHL to DHL. This review distills tools that have been used for older adults. No assessment tools have been identified that only target older adults for DHL (Table 2).

**Table 2:** DHL assessment tools for older adults.

**Name**	**Author Year**	**Theories/** **sources based on**	**Scoring method**	**Content**	**Study in older adults**		
					**Author**	**Year**	**Country**
eHEALS	Norman and Skinner (5)	Lily model	5-point Likert scale	**Items:** Q1: I know how to find helpful health resources on the Internet Q2: I know how to use the Internet to answer my health questions Q3: I know what health resources are available on the Internet Q4: I know where to find helpful health resources on the Internet Q5: I know how to use the health information I find on the Internet to help me Q6: I have the skills I need to evaluate the health resources I find on the Internet Q7: I can tell high quality from low quality health resources on the Internet Q8: I feel confident in using information from the Internet to make health decisions	Kim H, Yang E, Ryu H, et al.	2021	South Korea
					Baek JJH, Soares GH, da Rosa GC, et al.	2021	Brazil
					He Y, Guo L, Zauszniewski JA, et al.	2021	China
					Lin C-Y, Brostrom A, Griffiths MD, et al.	2020	Iran
					Zrubka Z, Hajdu O, Rencz F, et al.	2019	Hungary
					Duplaga M, Sobecka K, Wojcik S	2019	Poland
					Stellefson M, Paige SR, Tennant B, et al.	2017	United States
					Aponte J, Nokes KM	2017	Spain
					Sudbury-Riley L, FitzPatrick M, Schulz PJ	2017	United States, United Kingdom, and New Zealand
					Chung SY, Nahm ES	2015	United States
e-HLS	Seçkin et al. (40)	Internet survey; Lecture review.	5-point Likert scale	**It is a 19-item self-report scale that examines the behavioral, communicational, and attitudinal components of health literacy among ehealth information seekers**. (1) read disclosure statements on health websites. (2) check for credentials and institutional affiliations of those who provide information on websites. (3) check the ownership of a health website, (4) check a website's sponsor(s). (5) check for financial ties between website information and the website's sponsor(s). (6) appraise the adequacy and integrity of information providers' credentials. (7) check to see whether a physical address is provided. (8) check for stated goals and objectives. (9) appraise whether coverage of health topics is clear and comprehensive. (10) check whether other print or Web resources confirm information provided. (11) checked whether information is current and updated. (12) check for the last time information was updated. (13) if they were confident in their ability to appraise information quality on the Internet, and if they (14) asked health professionals for advice about where to find credible information on the Internet. (15) discussed information obtained from the Internet with a health professional. (16) believed information provided on the Internet was credible. (17) believed information provided on the Internet was balanced and accurate. (18) thought information provided on the Internet was the same as or better than what most health professionals provided, and (19) trusted the Internet for obtaining accurate health information.	Seckin G, Yeatts D, Hughes S, et al.	2016	United States
					Kim H, Yang E, Ryu H, et al.	2021	South Korea
DHLI	Van der Vaart and Drossaert. (11)	web1.0 web2.0	4-point Likert scale, fictional situation	Items: How easy or difficult is it for you to… 1. Use the keyboard of a computer (eg, to type words)? 2. Use the mouse (eg, to put the cursor in the right field or to click)? 3. Use the buttons or links and hyperlinks on websites? When you search the Internet for information on health, how easy or difficult is it for you to… 4. Make a choice from all the information you find? 5. Use the proper words or search query to find the information you are looking for? 6. Find the exact information you are looking for? 7. Decide whether the information is reliable or not? 8. Decide whether the information is written with commercial interests (eg, by people trying to sell a product)? 9. Check different websites to see whether they provide the same information? 10. Decide if the information you found is applicable to you? 11. Apply the information you found in your daily life? 12. Use the information you found to make decisions about your health (eg, on nutrition, medication or to decide whether to ask a doctor's opinion)? When you search the Internet for health information, how often does it happen that… 13. You lose track of where you are on a website or the Internet? 14. You do not know how to return to a previous page? 15. You click on something and get to see something different than you expected?			
				When typing a message (eg, to your doctor, on a forum, or on social media such as Facebook or Twitter) how easy or difficult is it for you to… 16. Clearly formulate your question or health-related worry? 17. Express your opinion, thoughts, or feelings in writing? 18. Write your message as such, for people to understand exactly what you mean? When you post a message on a public forum or social media, how often… 19. Do you find it difficult to judge who can read along? 20. Do you (intentionally or unintentionally) share your own private information (eg, name or address)? 21. Do you (intentionally or unintentionally) share some else's private information?	van der Vaart R, Drossaert C	2017	Holland
					Cheng C, Elsworth G, Osborne RH	2021	Australia
eHLQ	Lars Kayser et al. (45)	eHLF	4-point Likert scale,	**Scales (7 to 9 items per scale)** 1. Using technology to process health information 2. Understanding of health concepts and language 3. Ability to actively engage with digital services 4. Feel safe and in control 5.Motivated to engage with digital services 6.Access to digital services that work 7. Digital services that suit individual needs	Liu P, Yeh L-L, Wang J-Y, et al.	2020	Taiwan, China
DHLA	Liu et al. (46)	eHEALS	5-point Likert scale	**Items:** 1. My ability to use computer/smartphone to find information that I need on the internet 2. My ability to find health- or disease-related information on the internet 3. My ability to find information on internet to understand health problems or diseases 4. My ability to find information on the internet to answer the questions on health care or disease treatment 5. My ability to use information found on the internet to discuss with health care professionals 6. My ability to judge whether the health care information found on the internet is accurate or not 7. Beliefs about the health care information that I find on the internet 8. Beliefs about the health care information provided by physicians that I find on the internet 9. Beliefs about the health care information provided by hospitals that I find on the internet 10. Beliefs about the health care information based on folklore and customs that I find on the internet	Liu P, Yeh L-L, Wang J-Y, et al.	2020	Taiwan, China

#### eHealth literacy scale (eHEALS)

The eHEALS (5) is a scale designed to measure an individual's combined knowledge, comfort, and perceptual skills in discovering, evaluating, and applying ehealth information to address health problems. Norman and Skinner compiled it based on their Lily Model of eHL (5). The scale's Cronbach α was 0.88. The correlation coefficient ranged from r = 0.51 to 0.76, and from baseline to 6-month follow-up, the test-retest reliability ranged from 0.68–0.40. At present, local translation, reliability, and validity studies of the scale have been conducted among older adults in many countries, proving that eHEALS is a reliable and effective tool for older adults (47–55). Although it is the most widely used DHL assessment tool so far, eHEALS is a self-assessment scale and lacks objective items for evaluating the DHL of older adults. In the face of the great changes in health knowledge acquisition and communication induced by today's digital environment, eHEALS' existing projects are insufficient.

#### Electronic health literacy scale (e-HLS)

e-HLS is a self-assessment scale developed by Seckin et al. (56). Through exploratory factor analysis and confirmatory factor analysis, for the older subsample (age ≥ 60 years) the Cronbach α = 0.94, CFI = 0.95, NFI = 0.90. e-HLS evaluates the six competencies mentioned in the traditional concept of e-health literacy while paying attention to the expansion of the concept of eHL, by adding indicators for evaluation, communication, and use of e-health information. It contains tools in three domains: behavioral literacy (action factor), cognitive literacy (trust factor), and interaction literacy (communication factor) (56). He et al. examined the reliability and validity of e-HLS among patients with stroke in China. They found that e-HLS can be applied to these patients after translation and cultural adaption (Cronbach'α = 0.907, test-retest reliability = 0.691) (57).

#### Digital health literacy scale (DHLI)

Van der Vaart and Drossaert developed DHLI in 2017 to measure navigation skills, operational skills, evaluating reliability, information searching, and determining relevance. Based on eHEALS, the evaluation of information communication and privacy confidentiality ability was included (11). Additionally, the researchers added a simulated situational assessment for each skill. In a study among older Korean adults, the Cronbach α = 0.93, and test-retest reliability was 0.844. Findings suggest that K-DHLI is reliable and effective for evaluating the use of e-health resources in older adults (47).

#### The eHealth literacy questionnaire (eHLQ)

eHLQ is a self-assessment evaluation tool developed by Kayser et al. (45). It is based on the Ehealth Literacy Framework(eHLF), which comprises seven dimensions that describe the attributes of the users, the intersection between users and the technologies, and users' experience of systems (45, 58). After using the Bayesian mediated multiple indicators and cause models, Cheng's research demonstrates that eHLQ can be used to access valuable suggestions, help optimize digital health use, and promote health equity (59).

#### Digital health literacy assessment (DHLA)

DHLA was developed by Liu et al. (46). The researchers considered that the environment and culture also impact DHL, and therefore, based on eHEALS, items related to these factors were included. It is a self-assessment tool that can classify participants into high, moderate, and low-risk groups based on the degree of risk of misinterpreting health information. The internal consistency of DHLA was satisfactory (α = 0.87), and the construct validity factor analysis found three factors, accounting for 76.6% of the variance. Studies have not yet been conducted with older adults in other countries (46).

### Intervention method

The existing DHL intervention methods for older adults are primarily based on education and training. Usually, they adopt the Health Belief Model and the Information-Motivation-Behavioral skills model as the conceptual framework (60–63). Of the included studies, three entailed face-to-face teaching. Chang et al. conducted a structured curriculum in community activity centers for older adults. The course content was practical and effectively improved their DHL (61). However, the training process did not vary according to individuals, and the training interface did not fully consider the needs of older adults (such as fonts and article length). Lee and Kim adopted an intergenerational mentoring approach, allowing college students to provide face-to-face mentoring to older adults, ensuring that older adults have a pleasant learning experience while also addressing their problems in a targeted manner (64). However, they included a small sample and did not explicitly assess what older adults learned. Xie reduced the computer anxiety of older adults and increased their interest in health knowledge by allowing them to conduct unified training in the library (65). However, the study did not have a control group and could not prove whether the intervention was scientifically effective. Of the included studies, four studies adopted online interventions. Prior to the intervention, older adults' DHL status and training needs were established in advance through focus group discussions or questionnaires. Fink et al. and Nahm et al. have designed courses that can improve older adults' digital health knowledge and digital equipment operation skills (62, 63); The course designed by Perestelo-Perez et al. not only provides reading materials, videos, and online exams but also allows older adults to communicate with each other in the learning process (60). Bevilacqua et al. used a training called ACCESS program, which was based on blended didactical and interactive educational techniques (66). It enables older adults to gradually master the concept of digital health by using related applications, and adopting digital health equipment or software to communicate in five-stage courses. In the context of the ongoing COVID-19 pandemic, online teaching is more conducive to preventing the spread of the virus. However, online education requires certain resources (such as computers and mobile phones); therefore, online education is inaccessible to older adults who do not have or use such equipment (Table 3).

**Table 3:** Intervention methods for improving older adults' DHL.

**Authors, publication year, country**	**Study objective**	**Conceptual framework**	**Research method**	**Intervention methods**	**Population**		**Data collection** **method(s)**	**Results**
					**Intervention**	**Control**		
Xie (65) United States	To explore the effects of curriculum training on improving e-health literacy among older adults?	Not mentioned	Literature review, Questionnaire survey	The Helping Older Adults Search for Health Information Online: A Toolkit for Trainers tutorial developed by the National Institute on Aging (NIA) of the NIH was used as the curriculum. The freely available Toolkit aims at improving older adults' ability to seek, find, and understand health information from NIH Senior Health and MedlinePlus, and to apply the knowledge gained to addressing or solving a health problem of personal interest. It includes detailed lesson plans, interactive in-class exercises, take home practice exercises, and other supportive handouts.	Older adults aged 60–89 (*N* = 218)	/	Questionnaires: Computer Anxiety Scale, Attitudes Toward Computers Questionnaire	Computer and Web knowledge significantly improved from pre- to post-intervention and computer attitudes significantly improved. Anxiety significantly decreased while interest and efficacy both increased.
Fink et al. (62) United States	Assessing the feasibility of creating a website “Your Health Online” to improve older adults' skills in identifying high-quality online health information	Health Belief Model (HBM)	Semi-structured interview method, Questionnaire survey, Quasi-experimental research	64 participants were randomly assigned to Your Health Online: Guiding eSearches or to an analogous slide-based-tutorial and compared in their knowledge, self-efficacy, and program assessment.	Guiding eSearches (*N* = 36)	Evaluating online health information (*N* = 29)	Self-made questionnaire about evaluation, knowledge, self-efficacy, Internet use and program assessment	Experimental participants assigned significantly higher ratings of usability and learning to the new site than controls did to their tutorial although no differences were found in self-efficacy or knowledge.
Lee et al. (64) United States	1. IMU senior mentees demonstrate greater eHealth literacy, proactive attitude toward using HIT, and reduced technophobia. 2. IMU senior mentees experience a decrease in feelings of social isolation.	Adult-learning principle	Semi-structured interview method, Questionnaire survey	Using IMU to innovative intervention, offering educational opportunities for college students to interact with older adults in the classroom, research interviews at senior centers, and intergenerational exchanges *via* youth-led tutorials on using HIT and social networking services	Average age = 73.82 (*N* = 55)	/	Questionnaires: eHEALS, Computer Efficacy subscale, the Attitudes Toward Computers Questionnaire, Computer Anxiety Scale, Computer Attitude Scale	Older adults presented significant improvement between pre- and post-surveys in various outcomes such as eHealth literacy, technophobia, self-efficacy, and interest in technology. Intergenerational interaction brought about by IMU helped to decrease social isolation among older adults. Qualitative data revealed that individualized training, modifications, adaptations, and intergenerational interactions can decrease their anxiety and boost their confidence.
Nahm et al. (63) United States	Assess the impact of an older adult friendly Theory-based Patient portal e-Learning Program (T-PeP) on PP knowledge, selected health outcomes (health decision-making self-efficacy [SE] and health communication), PP SE and use, and e-health literacy in older adults.	Health decision-making self-efficacy	Two-arm parallel-group randomized controlled trial; Questionnaire survey	The 3-week T-PeP was developed for older adults to learn to use PPs to manage their health. T-PeP includes learning modules, moderated discussion boards, and a virtual library (VL). A discussion forum accompanied each module, and trained nurse moderators facilitated the discussions.				
				Intervention group was encouraged to visit the T-PeP website at least once a week to review the new module and share their thoughts and experiences on the discussion boards. The nurse moderator facilitated the discussions and monitored them daily to identify any untoward postings. Participants could further explore PP-related topics and selected health information using the VLs. No specific intervention was provided to the control group participants.	Age: 69.7 ± 8.6 (*N* = 138)	Age, M ± SD 70.4 (8.5) (*N* = 134)	Questionnaires: eHEALS, Computer-Based Personal Health Record (PHR) scale, Decision Self-Efficacy Scale, Components of Primary Care Index and self-made questionnaire	At 3 weeks, the intervention group showed significantly greater improvement than the control group in all outcomes except PP use. At 4 months, the intervention effects decreased, but PP SE remained significant, and the intervention group showed higher frequency of PP use than the control group. The study findings showed that the T-PeP was effective in improving selected health and PP usage outcomes.
Perestelo-Perez et al. (60) 7 countries in Europe	To develop a series of massive open online courses (MOOCs) to improve the DHL skills of European citizens.	DigComp (European Digital Competence Framework)	Literature Review, Exploratory survey; Focus groups and group interviews, Questionnaire survey	MOOCs are a curriculum model that uses traditional learning methods (reading materials, videos, online exams), as well as interactive components such as user forums and discussions, to facilitate interaction among participants, facilitators, and experts. The MOOCs developed by the researchers focus on four essential elements for developing DHL's digital capabilities: discovering, understanding, and evaluating electronic resources and applying this knowledge to address health problems.	Over 60 years old (*N* = 390)	/	Questionnaires: eHEALS and self-made questionnaire	MOOCs can be an effective educational resource for DHL and a facilitator of shared decision-making processes.
Bevilacqua et al. (66) Italy	1. Exploring, implementing, and evaluating new modes of socially embedded learning opportunities for older adults with low technical skills, 2. Identifying ways to improve digital literacy in regard of internet skills and the everyday usage of assistive technologies in older individuals, 3. Fostering a new learning culture for later-life learning.	Blended didactical, Interactive educational techniques	Questionnaire survey, Self-contrast before and after	The training is divided on the following modules: 1. Raising awareness of eHealth and health literacy: introduction to the aims and context of the ACCESS project, Achieving new knowledge: introduction to Health and eHealth literacy, 2. Practicing new skills: digital health apps and skills with practice session, 3. Practicing new skills: usability with practice session, 4. Social communication: inter-generational mode, 5. Self-evaluation and sustainability of the improvement (final questionnaire).	Age, mean ± SD (range) 68.2 ± 5.0 (50–77)	/	Questionnaires: eHEALS and Survey of Technology Use (SOTU)	The results showed a statistically significant difference between the eHealth Literacy Scale (eHEALS) mean value before and after the course. A significant negative correlation was found between eHEALS and positive/total Survey of Technology Use (SOTU). There is a strong positive and statistically significant relationship between satisfaction with the training and eHEALS. The results indicate that the intervention increased the digital competences of participants connected to health.
Chang et al. (14) Korea	To verify the feasibility and preliminary effects of a behavior theory-based education program designed to improve older adults' ability to search for, understand, and use internet-based health information.	The information- motivation-behavioral skills (IMB) model	Single-group pretest-posttest design, Questionnaire survey	Each class had a structured curriculum consisting of four parts: 20 min for the introduction and previous session review, 65 min for development, 15 min for consolidation, and a total of 20 min for break time (including individual supplementary educational time for was anyone who needed it). The student-to-instructor ratio was slightly <4:1. The subjects covered in the classes were as follows: Week 1, computer basics, week 2, understanding of the NHIP website, week 3, use of the NHIP website, week 4, use of the NAVER portal, week 5, evaluating the credibility of online health information.	Mean age was 74 years (age range 67–87) (*N* = 11)	/	Questionnaires: Self-reported questionnaire, Computer/Web knowledge questionnaire, Technology Acceptance Model 3 scale	Computer/Web knowledge, attitude toward internet-based health information, eHealth literacy score, searching performance scores, and level of understanding of internet-based health information —showed significant improvement immediately after the intervention. This pilot study reveals that a behavior theory-based education program for utilizing internet-based health information is an effective way to increase older adults' eHealth literacy.

## Discussion

The primary factors that affect the DHL of older adults include demographic and sociological factors, whether or not they use digital devices, confidence in managing health through digital devices, attitudes, and family support. Older people with better social conditions and higher education are more likely to accept digital technology to manage their health. Attitudes toward digital health and digital devices will also affect their use. Meanwhile, the DHL of older adults will directly or indirectly affect their quality of life, life satisfaction, anxiety, and other factors. Notably, all of the included cross-sectional studies employ the concept of eHL regardless of the year in which the study was conducted. In future research on older adults, researchers should introduce the concept of DHL and corresponding evaluation tools to explore the influencing factors of older adults' DHL. The demographic and socioeconomic characteristics of older adults should be further split to obtain more detailed information on the factors related to DHL. More in-depth research will help better understand the current situation of DHL in older adults to facilitate improvement in their DHL in a targeted manner.

Among the assessment tools used to evaluate the DHL of older adults, eHEALS has been widely used in research in many countries over the past 5 years, and is a popular assessment tool. Except for DHLI, which employs virtual situations to evaluate the simulated operation of older adults, the others use self-evaluation, which may lack objectivity. Furthermore, all research tools included in this review have only been adaptively tested in older adults and were not developed to validate DHL in older adults specifically. In the future, researchers that develop assessment tools should be aware that the concept of DHL is constantly evolving, and the competencies it requires from individuals are different from those of eHL. In addition to evaluating the ability to obtain, evaluate, and apply health information, the ability to communicate, integrate health information, and protect privacy in the process should also be evaluated. In addition, to evaluate the DHL of older adults more accurately, researchers may consider developing an evaluation tool applied especially to older adults. Moreover, they should attempt to avoid the questionnaire method owing to the lack of objectivity in the evaluation results.

In general, the existing intervention methods to improve the DHL of older adults have achieved certain results such as improving the eHEALS score, strengthening the computer application ability of older adults, and enhancing the confidence of older adults in the application of digital devices. In the future, in addition to the design of intervention methods, researchers should consider the following aspects: Prior to training, older adults should be apprised of the benefits of digital technology. Researchers should mobilize older adults' enthusiasm to learn to enhance their confidence in health management through digital technology. Additionally, it is important to identify the possibility of implementing group teaching for older adults with different digital abilities. During the training process, researchers should also pay attention to the continuous decline of older adults' cognitive and physical functions (67). Researchers should revise training plans dynamically while accurately assessing the training needs of older adults and adding customisations based on individual preferences. After the initial stages of the COVID-19 pandemic, the digital health teaching method for older adults may change. The previous face-to-face teaching method may not have been carried out effectively owing to pandemic-related restrictions. If an online course approach is adopted, it will create another obstacle for older adults. Further teaching methods should be developed in the future exploration of teaching practice, such as family collaboration mode and mutual aid mode.

### Limitations

Although four commonly used databases were applied for the literature search, studies on older adults' DHL in other databases may have been excluded. Additionally, the title and abstract review may be insufficient to reflect the initial findings of all studies effectively, and some relevant articles may have been removed. Finally, only Chinese and English literature were selected in this review process, which may result in an incomplete literature search.

## Conclusion

The results of this scoping review reflect the current state of research on DHL in older adults. In general, among the included studies, not many studies focus solely on DHL, and most studies still focus on eHL or consider eHL and DHL as the same. However, although in recent years digital technologies have been increasingly applied to older adults' health care, the capabilities contained in the conceptual framework of eHL have been unable to cope with it effectively. Therefore, to ensure equitable and inclusive access to health knowledge in the digital healthcare era, DHL for older adults needs to be improved, as this attempt can bridge the digital divide and improve health equity. In the future, studies are required for comprehensive and in-depth exploration of DHL of older adults.

## Author contributions

XW and WL contributed to the literature review and classification. XW wrote the first draft of the manuscript. WL contributed to manuscript revision and approved the submitted version. All authors contributed to the article and approved the submitted version.

## Funding

This study has been funded by Shanghai Municipal Health Commission (202150032), Shanghai Hospital Association (X2020083), and Shanghai Jiao Tong University (ZT201902).

## Conflict of interest

The authors declare that the research was conducted in the absence of any commercial or financial relationships that could be construed as a potential conflict of interest.

## Publisher's note

All claims expressed in this article are solely those of the authors and do not necessarily represent those of their affiliated organizations, or those of the publisher, the editors and the reviewers. Any product that may be evaluated in this article, or claim that may be made by its manufacturer, is not guaranteed or endorsed by the publisher.
